# Wnt/β-catenin signaling in cancers and targeted therapies

**DOI:** 10.1038/s41392-021-00701-5

**Published:** 2021-08-30

**Authors:** Fanyuan Yu, Changhao Yu, Feifei Li, Yanqin Zuo, Yitian Wang, Lin Yao, Chenzhou Wu, Chenglin Wang, Ling Ye

**Affiliations:** 1 State Key Laboratory of Oral Diseases & National Clinical Research Center for Oral Diseases, West China Hospital of Stomatology, Sichuan University, Chengdu, China; 2 Department of Endodontics, West China Stomatology Hospital, Sichuan University, Chengdu, China; 3 Department of Head and Neck Oncology, West China Hospital of Stomatology, Sichuan University, Chengdu, China

**Keywords:** Molecular biology, Cell biology, Biochemistry

## Abstract

Wnt/β-catenin signaling has been broadly implicated in human cancers and experimental cancer models of animals. Aberrant activation of Wnt/β-catenin signaling is tightly linked with the increment of prevalence, advancement of malignant progression, development of poor prognostics, and even ascendence of the cancer-associated mortality. Early experimental investigations have proposed the theoretical potential that efficient repression of this signaling might provide promising therapeutic choices in managing various types of cancers. Up to date, many therapies targeting Wnt/β-catenin signaling in cancers have been developed, which is assumed to endow clinicians with new opportunities of developing more satisfactory and precise remedies for cancer patients with aberrant Wnt/β-catenin signaling. However, current facts indicate that the clinical translations of Wnt/β-catenin signaling-dependent targeted therapies have faced un-neglectable crises and challenges. Therefore, in this study, we systematically reviewed the most updated knowledge of Wnt/β-catenin signaling in cancers and relatively targeted therapies to generate a clearer and more accurate awareness of both the developmental stage and underlying limitations of Wnt/β-catenin-targeted therapies in cancers. Insights of this study will help readers better understand the roles of Wnt/β-catenin signaling in cancers and provide insights to acknowledge the current opportunities and challenges of targeting this signaling in cancers.

## Introduction

As an evolutionarily conserved signaling that governs numerously vital embryonic and somatic processes, such as cell fate determination, organogenesis, tissue homeostasis, and a variety of pathological conditions, Wnt/β-catenin signaling also plays crucial roles in cancers.^1^ Aberrant Wnt/β-catenin signaling has been uncovered to be tightly woven with many aspects of cancers, including the onset, progression, malignant transformation, and so on.^2,3^ Evidence-based medicine has further proved that the abnormal activation of this signaling showed non-negligible effects on cancer-associated mortality.^4–6^ Despite these broad acknowledgments of the significant impact of Wnt/β-catenin signaling on cancers, advances in targeted therapies remain largely incipient. Considering the extremely heavy clinical burden of Wnt/β-catenin-associated cancers globally, it is urgent to comprehensively summarize the up-to-date knowledge of Wnt/β-catenin signaling in cancers and to clarify the current status and challenges of developing Wnt/β-catenin-associated targeted therapies.

Collectively, it is necessary to systematically review the experimental and clinical knowledge of Wnt/β-catenin signaling in cancers and present the status quo of Wnt/β-catenin-targeted therapies in cancers. With the rapid development of modern pharmacology and evidence-based medicine, certain approaches of targeting Wnt/β-catenin signaling in cancers have already achieved the path of clinical trials. These promising advances will endow researchers and clinicians with more choices and fundaments to better manage aberrant Wnt/β-catenin-associated cancers. In this article, we have systematically reviewed the most updated knowledge of Wnt/β-catenin signaling in caners and targeted therapies in accordance. Therefore, we sought to provide readers with the latest progress of Wnt/β-catenin signaling in cancers and demonstrate both opportunities and challenges of Wnt/β-catenin signaling-dependent targeted therapies in cancers.

## Brief intro of Wnt/β-catenin

The nomination of Wnt is after wingless in drosophila and int1 in mammalians.^7,8^ As the prerequisite of understanding the roles of Wnt/β-catenin in cancer, in this part, we will briefly introduce the signaling transduction. Numerous researches have already depicted an integral scene of the core components of β-catenin-dependent Wnt signaling. It consists of extracellular ligands, agonists; trans-membraned receptors/co-receptors; intracellular compounds including disheveled (Dsh in drosophila and Dvl in mammals), degradation complex comprising glycogen synthase kinase 3 β (Gsk3β), casein kinase1α (CK1α), Axin/conductin, and adenomatous polyposis coli (Apc), β-catenin, and transcription factors.^9^

Extracellular Wnt ligands are inevitable to switch-on downstream signaling cascade. The extracellular secretion and functions of Wnt ligands depend on post-transcriptional modifications.^10^ The post-transcriptional modifications of Wnt ligands mainly include glycosylation, palmitoylation, and acylation.^11–13^ Especially, the acylation is necessary for extracellular transport and receptor/co-receptors recognition and bounding.^14,15^

With respect to the receptor/co-receptors of Wnt/β-catenin signaling, including frizzled (Fzd) receptors and Lrp co-receptors. For Fzds family, it owns at least 10 members of G protein-coupled receptor (GPCR).^16^ The highly conserved cysteine-rich domain (CRD) of Fzds manipulates the ligand recognition and binding.^15,17^ In addition, as co-receptors to Wnt ligands, Lrps consist of Lrp5 and 6, whose extracellular domains interact with Fzds and then its intracellular domains trigger further signaling transduction.^18,19^ In addition to receptor/co-receptors, there also exist certain extracellular regulators that can influence the ligand–receptor/co-receptors interaction. For instance, the R-spondins (Rspo), member 1/2/3/4,^20^ which coordinates with leucine-rich repeat-containing GPCR (Lgr) 4/5/6 to enhance Wnt/β-catenin signaling.^21–24^ Specifically, the Rspo-Lgr complex increases Lrp5/6 phosphorylation and inactivates Wnt repressors Rnf43 and Znrf3.^23,25,26^ Rnf43 and Znrf3 are E3 ubiquitin ligases that mediate the degradation of Fzds.^26,27^ Furthermore, certain intracellular regulators can also abolish the signaling cascade downstream of ligand–receptor interaction. Dsh, a cytoplasmic protein with three highly conserved sections,^28^ directly interacts with the C-terminal of Fzds via its PDZ region.^29,30^

Finally, after receiving the upstream activation signals, β-catenin functions as the ultimate effector.^31,32^ Without canonical Wnt ligands, β-catenin binds to cadherin of cytoplasmic sides rather than being transported into the nucleus, further phosphorylated and eliminated by degradation complex.^32–34^ Phosphorylated β-catenin is degraded by the ubiquitin-proteasome system to keep the low level of free β-catenin in the cytoplasm.^32,33^ Conversely, once being activated, intracellular β-catenin is rapidly enriched, and then trans-localized into the nucleus to regulate gene expressions.^32,33^ Apart from directly manipulating gene transcription as a TF, β-catenin can also form a transcriptional complex with Lef/Tcf via its armadillo repeats regions.^32,35^ Intriguingly, the degradation complex of β-catenin is concise and complicated. As soon as the degradation complex converts into the active form, it phosphorylates β-catenin for ubiquitination, which sends it to the proteasome. When the degradation complex is deactivated, β-catenin accumulates and influxes into the nucleus to initiate the transcription of downstream genes.

## Wnt/β-catenin signaling in cancers

Basing on the subtle summary of Wnt/β-catenin signaling, in this part, we aimed to further demonstrate the roles of Wnt/β-catenin signaling in cancers. Specifically, we will follow the progressively organized structure going behind the signal conduction cascade of this pathway: extracellular, membrane-linked, and intracellular (cytoplasmatic and nuclear) compositions.

## Extracellular compositions

### Porcupine (Porc)

**Fig. 1:**
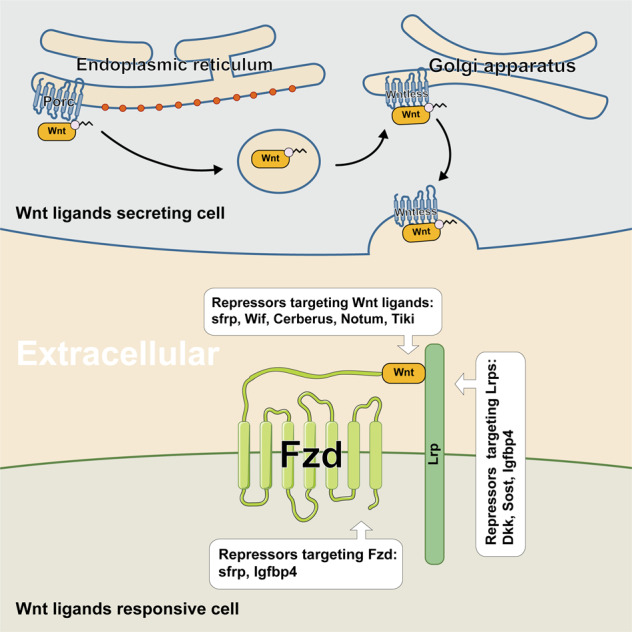
The extracellular components and signaling transduction of Wnt/β-catenin signaling. In this figure, we do not distinguish the autocrine or paracrine patterns of Wnt ligands

Porc is a membrane-bound *O*-acyltransferase (MBOAT), by whose palmitoylation function Wnt ligands can be subsequently secreted and recognized.^36^ After being palmitoylated, Wnt ligands will bind to wntless and be transported into cell membrane from Golgi apparatus^37^ (Fig. 1). Thereout repressing Porc is a candidate way against tumors with aberrant Wnt/β-catenin activation. Nowadays, inhibitors targeting Porc have been uncovered to be underlyingly beneficial for diverse types of cancers.^38^

### Ligands

The Fzds are responsible for recognizing and receiving canonical Wnt ligands, which are defined as β-catenin-dependent ligands. In this review, we focus on the canonical Wnt ligands, including Wnt1, 2, 3, and 3a.

#### Wnt1

Previous studies have shown that knockout of wg, the homolog gene of mammalian Wnt1, caused the wingless phenotype in *Drosophila*.^7,8^ Experimental and clinical analysis further revealed the frequent abnormal upregulations of Wnt1 in massive cancers.^39–42^ Abnormal Wnt1 expressing patients comprised the majority of cancer patients of non-small cell lung cancer (NSCLC).^43^ Nevertheless, a Wnt1-dependent gene, Wnt1-inducible signaling pathway protein 2 (WISP2), was reported to effectively undermine the immunologic evasion of cancer cells.^39,44^ In addition to the direct involvements of Wnt1 in cancers, certain upstream modulators of Wnt1 can repress the viability of a few sorts of cancer cells, containing RU484, MicroRNA-140-5p, and SJ26.^45–47^

Encouragingly, inhibiting Wnt1 relieved the growth and progression of breast cancer in a transgenic murine model. Conversely, overexpression of Wnt1 promoted the growth of cancers.^48,49^ Therapeutically, Blocking Wnt1 has been found to strengthen the apoptosis of colorectal cancer (CRC) cells via the utilization of Wif1, Wnt1-specific siRNAs, and neutralizing antibodies.^40^

#### Wnt2

Similarly, the overexpression of Wnt2 has also been detected in human fibroadenomas, breast cancer, pancreatic cancer, and CRC.^50–53^ Despite the concerns that in CRC (CRC) Wnt2 perhaps accelerated its migration and invasion,^52,54^ on the contrary, in other kinds of cancers such as gastric, pancreatic, and NSCLC, Wnt2 aggregates the cancer progression.^55–57^ With respect to its therapeutic potentials, direct silence of Wnt2 alleviated the xenograft breast cancer growth and rescued the malignance and the chemo-drugs resistance in breast cancers.^58,59^

#### Wnt3

Wnt3 is a homologous gene of Wnt3a, and the similarity is up to 84.2% total amino-acid identity in humans.^60^ The level of Wnt3 mRNA was obviously upregulated in primary breast and rectal cancer.^60^ Wnt3 plays a cacoethic role in multiple cancers, such as CRC,^61^ breast cancer,^62,63^ NSCLC,^64,65^ and prostate cancer.^66^ Specifically, the excitation of Wnt3 speeds up the tumorigenesis of CRC.^61^ Furthermore, it has been implied to elevate the epithelium–mesenchyme transit (EMT) of breast cancers through Wnt/β-catenin.^62,63^ In terms of therapeutic choices, the downregulation relieved the progression of CRC by reducing cancer cell proliferation and migration.^61^ For NSCLC, knockdown of Wnt3 could increase drug sensitivity.^64,65^ In prostate cancer, inhibiting Wnt3 signaling by the deficiency of trophinin-associated protein attenuated the cancer growth.^66^ The demethylation of non-coding RNA miR-1247-5p provided an optional remedy for human hepatocellular carcinoma (HCC) via inhibiting Wnt3.^67^

#### Wnt3a

As the strongest Wnt/β-catenin stimulator, Wnt3a has been proposed to participate in numerous cancers. For instance, in most solid tumors, Wnt3a promoted the tumorigenesis and progression of CRC, prostate, liver, and lung cancers.^68–71^ Mechanistically, Wnt3a enhanced cancer cells proliferation, differentiation, migration, and self-renewal,^72–74^ and conversely inhibits cell apoptosis depending on activating Wnt/β-catenin.^68^ In leukemia, a study indicated that Wnt3a, activating Wnt/β-catenin, suppressed the proliferation of cancer cells.^75^

Therapeutically, in prostate cancers, targeting Wnt3a through Traf6 either Tmem64 restrained tumor development.^76,77^ For liver cancers, targeting Wnt3a by miRNA-195 and miRNA‑214 presented the possibility of cancer management.^78,79^

## Membrane-linked compositions

### Receptors and co-receptors

#### Fzds

Fzds, a subset of seven-transmembrane protein, are the principal receptors of canonical Wnt ligands.^80^ Fzds, mainly including subfamilies Fzd1/2/7, Fzd3/6, Fzd4, Fzd5/8, and Fzd9/10, are ubiquitously expressed in most animal species but not in plants and single-cell eukaryotes.^80^ The N-terminal CRD domain of Fzds spontaneously binds to Wnt ligands and Lrp5/6 co-receptors.^15,18^ The C-terminus of Fzds is localized in the cytosol which recruits and binds to Dsh to intracellularly trigger subsequent signal cascades.^81^ Fzds and Lrp5/6 are indispensable for Wnt/β-catenin activation, and non-eligibly, Fzds receptors, and Lrp5/6 co-receptors are both oncogenic under certain conditions.^82–85^ Except for main canonical Wnt ligands compromising Wnt1, 3, and 3a, there still exists several non-canonical Wnt ligands including Wnt5a, 7a, and 7b known to interact with Fzds under specialized circumstances.^86–88^

Fzd3 could be promotive for the development of Ewing sarcoma and breast cancer.^89–91^ Also, Fzd4 and Fzd5 were reported to be significantly increased in prostate cancer.^92,93^ Fzd6 highly emerged in CRC, breast cancer, and bladder cancer.^94–96^ Most importantly, Fzd7 is vitally essential in numerous cancers like HCC, breast cancer, gastric cancer, CRC and so on.^97^ Detailedly, Wnt3a-Fzd7 dependent Wnt/β-catenin signaling exaggerated the tumorigenesis and advancement of HCC.^98^ Prostate cancer metastasis is related to ERG-induced Fzd8 up-regulation.^99^ Besides, Fzd10 plays an important positive role in various cancers, at least in CRC development, but the level of Fzd10 is down in metastatic cancers.^100^ The Fzd10 expression may be a prognostic marker in CRC,^100^ and the same is true in synovial sarcoma.^101^

Next, the following part is about to concisely summarize the potential therapeutic values of targeting Fzds in cancers. Fzd1 is related to drug resistance in cancers, subsequently, inhibiting Fzd1 attenuating the resistance.^102–104^ Moreover, repressing Fzd1 via knocking down Fzd1, using rosiglitazone or miR-135b decreased the metastasis of breast cancer.^102–105^ For targeting Fzd3, by which some non-coding RNAs, like miRNA-505, miRNA-493, and HOXD cluster antisense RNA 1 relieve cancer progression.^106–108^ And Fzd7 deficiency is enough to combat Apc carcinogenesising.^84^ Fzd3 has a certain promotion function on Ewing sarcoma, breast cancer.^89–91^ Research shows that Fzd4 expression is extremely high in human prostate cancer cells and some non-coding RNA, like microRNA-505, microRNA-493, and HOXD cluster antisense RNA 1 (HOXD-AS1), relieves cancer progression by Fzd3.^92,106–108^ Fzd5 functions in prostate and gastric cancer,^93,94^ and Fzd6 in colorectal, breast, and bladder cancer.^94–96^ Fzd7 overexpression is detected in numerous cancers, like hepatocellular, breast, and CRC.^97^ WNT3A-Fzd7 interaction activates Wnt/β-catenin signaling in human hepatocellular carcinoma cells and promotes tumorigenesis.^98^ Fzd7 is vitally essential for the development of gastric cancer and it is the major receptor of Wnt ligands.^84^ Inhibitor targeting Fzd7 is effective for treating gastric cancer, no matter with Apc mutations or not.^84^ Fzd7 may be an extraordinarily effective therapy for gastric and CRCs.^84,109^ ERG is overexpression in most prostate cancer and Wnt/β-catenin signaling is activated.^99^ Prostate cancer metastasis is related to ERG inducing Fzd8 up-regulation.^99^ Besides, Fzd10 plays an important positive role in various cancers, at least in CRC development, but the level of Fzd10 is down in metastatic cancers.^100^ The Fzd10 expression may be a prognostic marker in CRC,^100^ and the same is true in synovial sarcoma.^101^

#### Lrps

As co-receptors of Wnt ligands Lrp5/6 are tightly associated with the growth of Wnt-hypersensitive tumors. The single-domain antibody fragments of Lrp5/6 can effectively relieve the development of intestinal cancers.^110^ Generally, the truncated LRP5 amplified Wnt/β-catenin signaling to severely promote the growth of parathyroid tumors.^111^ Herein, stabilizing of Lrp5 by Hsp90ab1 enhanced gastric cancer progression.^112^ Moreover, LRP6 also plays a positive role in various cancers, such as CRC, breast cancer, prostate cancer, and so on.^83,113–115^ Basing on these facts, LRP6 neutralizing antibodies were demonstrated to repress tumorigenesis.^116^ It is of note that contrarily targeting Lrp5/6 may be not eligible for managing metastasis of breast cancer.

## Extracellular repressors

### The abolishers of receptor/co-receptors

**Fig. 2:**
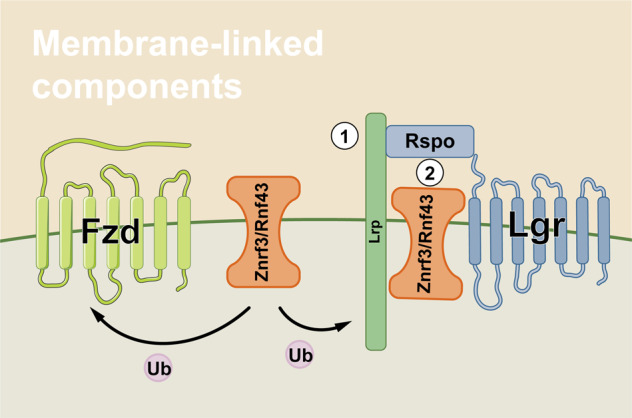
The membrane-linked components and signaling transduction of Wnt/β-catenin signaling. Ub ubiquitin, ① the switch-on of Fzd/Lrp ubiquitination, ② the switch-ff of Fzd/Lrp ubiquitination via Rspo function

Znrf3 and Rnf43, as transmembrane E3 ubiquitin ligases on cell surface, are negative regulators of Wnt/β-catenin signaling.^26,27^ Researches indicated that Znrf3/Rnf43 attenuated Wnt signaling by selectively ubiquitinating receptors/co-receptors of Wnts (Fzds and Lrps) to advance proteins degradation.^26,27^ Rspos, binding with Lgrs, can activate Wnt/β-catenin signaling by dually enhancing Lrp5/6 activity and removing Znrf3/Rnf43 from cell membrane^26,27^ (Fig. 2). Rspos could as well activate Wnt/β-catenin signaling via interacting with HSPGs independent of Lgrs.^117^ Interestingly, Rspo2 mutations were unraveled to associate with tetra-amelia syndrome, contributing to the destruction of Rspo2-Lgr binding.^118^ Current evidence depicted the crucial roles of somatic mutations of Rspos in cancers. In detail, Rspo fusions are regarded as important for tumorigenesis in CRC, by activating Wnt/β-catenin signaling.^119^ Rspo2/3 chromosome rearrangements can initiate and maintain tumor development absolutely through Wnt signals.^120^

Apart from Rspos, the mutations of Znrf3/Rnf43 were also proposed to participate in CRC. The most common mutations of Znrf3/Rnf43 were missense and truncating mutations, respectively.^121^ Znrf3 mutations were frequently detected in adrenocortical carcinoma, uterine corpus endometrial carcinoma, and skin cutaneous melanoma. Similarly, Rnf43 mutations were overall found in uterine corpus endometrial carcinoma, stomach adenocarcinoma, colorectal adenocarcinoma, ovarian cancer, and pancreatic ductal adenocarcinoma.^121,122^ Znrf3 and Rnf43 were not essential in the intestine but dysregulation of Znrf3/Rnf43 was important for the growth of CRC.^26,27,123^ Mutations of Rnf43, resulting in E3 ubiquitin ligases function loss, promoted CRC development and poor prognosis.^38^ Inactivating mutations of Rnf43 were related to Wnt dependency. LGK974, targeting on Wnt/β-catenin signaling, alleviated Rnf43 mutation-associated pancreatic cancer cell proliferation, however, which did not affect the non-mutant cancers.^124^ For CRC, Rspo/Rnf43 dysregulation plays a positive role in development and dominates over Znrf3.^125,126^ There still needs further researches to explore the relation between Wnt/β-catenin and Znrf3/Rnf43, as well as them in cancers.

#### The antagonists of receptor/co-receptors

Endogenous repressors of Wnt/β-catenin signaling can be divided into two groups: the reversible and the irreversible ones. The former group including competitively binds to the receptor/co-receptor to block the ligand–receptors interaction, like Dkks, Sost, sfrp, and so on. The latter group functions through different mechanisms. Notum deacetylates Wnts and permanently invalidates the recognition. Tikis hydrolyze Wnts to polymerize ligands and finally abolish ligand function. Considering that there are bare therapeutic analyses of targeting these irreversible repressors in cancer until now, in this part we will not further discuss relative aspects. (for more details, please find in Table 1)

**Table 1:** The mammalian endogenous repressors of Wnt/β-catenin signaling

Factors	Targets	Inhibitor/activator of the target	Mechanism	Reversible or irreversible	References
Dkks	Lrp	Inhibitor	Competitive combination	Reversible	^407,408^
Sfrp	Wnt, Fzd	Inhibitor	Competitive combination	Reversible	^409,410^
Wif	Wnt	Inhibitor	Competitive combination	Reversible	^411^
Sost	Lrp	Inhibitor	Competitive combination	Reversible	^412^
Cerberus	Wnt	inhibitor	Competitive combination	Reversible	^413^
Wise	LRP5/6	inhibitor	Competitive combination	Reversible	^414^
IGFBP-4	LRP6, Fzd	inhibitor	Competitive combination	Reversible	^415^
Wingful/Notum	Wnt	Inhibitor	Deacetylation, oxidization	Irreversible	^416^
Tiki	Wnt	Inhibitor	Cleavage, oxidization	Irreversible	^417^

## Intracellular compositions

### Adenomatous polyposis coli (Apc)

Apc gene locates on chromosome 5q21-q22, containing 8535 amino acids and encoding a cytoplasmic protein around 310 kDa. More than 1500 mutations of Apc were detected over multiple tumors, and majorly in CRC.^127^ Meanwhile, the most mutant sites were located on exon 15. Over 700 somatic mutations of Apc resulted in various types of cancers by truncating Apc protein that was dependent on nonsense (34%) or frameshift mutation (62%).^127,128^ Mutations of Apc can cause familial adenomatous polyposis (FAP), which is also the major hereditary carcinogenic factor in CRC progression.^129,130^ A study showed that around 72% of Apc mutations were detected spreading throughout the Apc gene in early-onset CRC.^131^

**Fig. 3:**
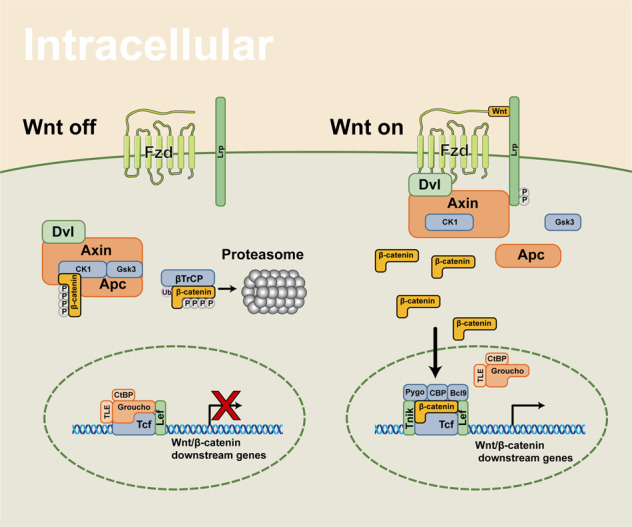
The intracellular components and signaling transduction of Wnt/β-catenin signaling

Apc gene has been identified as a vital suppressor in CRC genesis by inactivating Wnt/β-catenin signaling and stabilizing chromosomes. And mutations of Apc directly or indirectly lead to tumorigenesis as well.^132^ Mechanistically, Apc regulates β-catenin phosphorylation and restrains the nuclear trans-localization of β-catenin^95,133^ (Fig. 3). The three-hit hypothesis suggests that the abnormal mutation of Apc results in β-catenin abundance and abnormal activation of Wnt/β-catenin signaling in CRC.^134^ In contrast, the abnormal expression or truncation of Apc weakened the ability of tumor inhibition.^135^ Studies indicated that R2 and B motifs of Apc were the binding sites of Apc-Gsk3β/Axin, by which complex the diversity and structural stability of Axin were promoted.^60^ The loss-of-function (LoF) of Apc may be an essential contributor to various cancers, especially CRC.^136^ At present, experimental rodent models of CRC were initiated by knocking out of Apc.^137–139^

Apc also suppresses other cancers, such as lung, breast, gastric, and prostate cancers, excepting for CRC.^140–144^ The DNA methylation of Apc promoter is closely associated with various cancers, like lung cancer and prostatic cancer.^145,146^ The methylation reduces the normal expression of Apc in cancers, resulting in abnormal activation of the Wnt/β-catenin signaling pathway. Overall, this evidence indicated the repressive role of Apc in cancers, thus making it a promising way to remedy cancers via enhancing Apc function or restore the normal function of Apc.

#### Axin

Axin proteins, including Axin1 and Axin2, maintain β-catenin phosphorylation, thereby inhibiting signaling pathways by assembling the degradation complex with Gsk3β, Apc, and Ck1 (Fig. 3). Intriguingly, even though the Apc has been blocked, therapies targeting Axin could still be effective.^147,148^ Axin was identified as a suppressor in various cancers through majorly inhibiting Wnt/β-catenin signaling. Axins consist of three functional domains, the RGS domain of amino-terminal, the DIX domain of carboxyl-terminal, and the central region (AxinCR).^149^ Separately, the RGS domain is responsible for interacting with Apc to phosphorylate β-catenin.^150^ The DIX domain directly affects Dsh protein,^149,151^ and AxinCR binds to β-catenin and Gsk3β to regulate Wnt/β-catenin signaling.^33,149,152^

Some researches indicated that a single point mutation of Axin destroyed the stabilization of the RGS domain, resulting in Axin polymerization. The function of the Apc complex is affected by Axin self-polymerization. Inhibition of Axin and Apc complex together promoted tumor genesis and progression by enhancing Wnt/β-catenin signaling.^153,154^ Controlling Axin polymerization may be a potential therapeutic choice to suppress cancer development.^154,155^ Mutations of AxinCR were reported to accelerate the tumorigenesis in the following cancers: HCC, colorectal adenomas, ovarian carcinomas, lung carcinomas, and sporadic medulloblastomas. Therefore, erasing or eliminating the mutations of Axin could a promising method to combat diverse cancers.

#### Axin1/2 mutation in cancers

Axin1 mutations were detected widely in HCC.^156,157^ This mutation could phenocopy various tumors in animal models.^158,159^ Axin2 can partially compensate for the functional impairment caused by Axin1 mutation.^155,158,160^ Axin2 dysfunction was associated with a variety of tumors, including endometrial cancer,^161^ CRC,^162–165^ lung cancer,^166,167^ and breast cancer.^168–170^ In these cancers, Axin2 performed suppressive roles by mainly constraining the level of β-catenin.

#### Tankyrase (Tnks) in cancers

Tankyrase, consisting of two members (Tnks1 and Tnks2 in most species), participates in modulating Wnt/β-catenin signaling.^171^ Tankyrase1/2 is a poly (ADP-ribose) polymerase (PARP), attaching PAR chains onto substrates by its catalytic action.^172,173^ The enzymes bind and then PARylate Axins, utterly, the Axins are ubiquitylated and degraded.^174,175^ Additionally, researches showed that tankyrase enhanced Wnt/β-catenin signaling.^176,177^ A series of studies further proved that tankyrase could suppress cancer development via inhibiting the Wnt/β-catenin signaling pathway.^175,178–180^

#### Glycogen synthase kinase 3 (Gsk-3)

In Wnt/β-catenin signaling, generally, Gsk3 controls β-catenin degradation by comprising a degradation complex.^95,133,181^ Axin, phosphorylated by Gsk3, binds to and phosphorylates β-catenin, resulting in β-catenin degradation by ubiquitin proteasome.^95,133,181^ It is interesting that Gsk3 and CK1α can phosphorylate Wnt-co-receptor LRP6 to play positive roles in Wnt/β-catenin signaling activation.^182–185^ The dissociated Gsk3 from the degradation complex is essential for the nuclear shuttle of β-catenin^182–185^ (Fig. 3). So far, targeting Gsk3 may be a potential therapy for cancers. In certain cancers, Gsk3 may crosstalk with several pathways, including PI3K/PTEN/AKT/Gsk-3/mTORC1 and NF-κB pathway, and the details will be discussed in blow.^186–188^ Despite the function of Gsk3β is relatively clear in HCC, targeting Gsk3β in cancers still needs far more researches to elucidate its concise effects.

#### CK1

First encountered for phosphorylating casein in vitro, the CK1s are serine/threonine kinases that serve as pathway signal transductor in most eukaryotic cells. They are ubiquitously expressed in human tissues all through the developmental and adult period, and the Wnt/β-catenin signaling pathway is just one of the pathways that CK1s impact.^189^ The mechanisms of CK1 isoforms on regulating Wnt/β-catenin pathway are complicated. Depending on the substrates and subcellular location, CK1s play distinguished roles. To begin with, CK1α is the typical isoform for down-regulates Wnt/β-catenin signaling. As a composition of the degradation complex, CK1α initiates the phosphorylation of β-catenin.^190^ In fact, CK1δ/ε are also involved in this process by phosphorylating Apc (which strengthens the affinity of Apc to β-catenin), in collaboration with Gsk3.^34,191^ Furthermore, CK1ε phosphorylates and activities Dvl; however, this action also provokes negative feedback to inhibit Wnt/β-catenin pathway (vide infra).^192^ In terms of activation, on receiving Wnt ligands, firstly CK1ε phosphorylates Dvl, then CK1γ phosphorylates the cytoplasmatic domain of Lrp6, finally recruiting CK1α and Axin to bind with Lrp6 in the signalosomes,^193^ thus delivering the signal to downstream. Other mobilization effect includes the phosphorylation of CK1ε and CK1α to Tcf3 and Pygo, respectively.^194,195^

#### Dvl

The Dvls family is the transportation hub of Wnt/β-catenin signaling pathway. To adapt to this responsibility, the three highly evolutionally conserved domains of Dvls are the binding sites of various proteins. DIX, a highly conserved domain, is indispensable for the recognition of Axin.^196^ Moreover, DIX mediated the polymerization of Dvl monomer, which assembles as the anchoring site for Axin and Gsk3β.^197^ This process may have significant effects on the ligand–receptor/co-receptor internalization. CK1ε phosphorylating mediated DIX-E3 ligase interaction ubiquitinates Dvl, abolishing the polymerization of Dvls, ultimately inhibiting Wnt/β-catenin signaling.^192^ Next, the PDZ domain lying in the central part of Dvls, essential for the signal conduct from Fzd to downstream molecules, is also the most druggable region in Dvl. CK1 also targets this site.^190^ Near the C-terminal region is the DEP domain, whose role is still obscure in the canonical Wnt signaling pathway. Despite these three classic domains, Dvls also have a basic region and a proline-rich region, which may have effects in protein–protein interactions (PPI).^197^ Newish founds also indicated that Dvls shuttled between the membrane and the nucleus, and chances were that two distinct Dvls pools may exist.^198,199^ However, these discoveries were vague about the druggability of Dvl. So in this review, we suspended this topic aside.

#### β-catenin and Lef/Tcf in cancers

β-catenin is a multifunctional protein, which is versatile in various cellular events and human diseases. The very core of Wnt/β-catenin is the balance between the phosphorylation/dephosphorylation and degradation of β-catenin. In brief, the destruction complex phosphorylates β-catenin and to degradeβ-catenin by ubiquitin-proteasome system. There are some other kinases are associated with protein phosphorylation, including PP1, PP2A, and PP2C in Wnt/β-catenin signaling pathway. PP1 and PP2C play a positive role in Wnt/β-catenin signaling by dephosphorylating Axin.^200,201^ PP2A, a principal Ser/Thr phosphatase, involves multiple proteins phosphorylation, and importantly, the kinase malfunction could result in several cancers.^202–204^ PR55α, as a regulatory subunit of PP2A, can enhance the activity of Wnt/β-catenin signaling by regulating PP2A to suppress β-catenin phosphorylation.^205^ Hsp105, a PP2A regulator, is overexpressed in various tumors to reduce β-catenin degradation.^206^ Researches showed that PP2A dephosphorylated β-catenin to increase β-catenin accumulation.^205–207^ Therefore, it may be an alternative way to target PP2A in aberrant Wnt/β-catenin signaling cancers.

The stabilization of β-catenin is heavily associated with various cancers. In detail, the mutations of β-catenin are of great significance in tumorigenesis, progression, and prognosis of cancers.^208,209^ In blow, we will detailedly discuss the mutations of β-catenin in cancers. The constitutive activation form of β-catenin, the exon 3 mutations, is believed to regulate the genesis of hereditary non-polyposis CRC.^208^ In CRC, there are several mutations of β-catenin, leading to abnormally activated Wnt/β-catenin signaling.^209^ The mutations of β-catenin are commonly detected in HCC, uterine corpus endometrial carcinoma, adrenocortical carcinoma, and so on.^210–214^ The mutations of β-catenin majorly are missense that block Gsk3β consensus sites to activate Wnt/β-catenin signaling.^215,216^ Besides, β-catenin mutations may be a significant carcinogenic factor in endometrial carcinoma.^215,216^ Apart from the direct regulatory functions on tumorigenesis, the characteristics of β-catenin can also provide an approach to estimate the stage of low-graded, early-staged endometrial cancer recurrence.^215,216^

In addition to the direct transcription regulation of β-catenin as a transcription factor (TF), it can also form diverse types of transcription complex. β-catenin–Lef/Tcf includes Tcf/Lef, p300/CBP, and other proteins to assist β-catenin in binding to specific DNA sequence^217–219^ (Fig. 3). Tcf binds to Groucho/TLE, CtBP, and histone deacetylase proteins when β-catenin signaling is not activated.^217–219^ Tcf/Lef separates from Groucho/TLE and then composites β-catenin–Lef/Tcf complex, depending on X-linked inhibitor of apoptosis (XIAP) monoubiquitylating Groucho (Gro)/TLE.^220^ CBP promotes the transactivation of β-catenin/Tcf cooperating with thymine DNA glycosylase.^221^ In addition, the transcription complex recruits p300/CBP,^222,223^ Bcl9,^224^ Pontin522,^225^ Reptin52,^225^ Brg-1,^226,227^ Mllt/Af10-Dot1,^228^ SOX10,^229^ p68/p72,^230^ βTrCp1/Fbw1a,^231^ FOXM1^232–234^, and yes-associated protein 1 (YAP1).^235,236^

However, in CRC, HIFα can competitively bound to β-catenin to abolish the Tcf4/β-catenin complex, then enhancing the hypoxia tolerance of cancer cells to increase the survival in an anoxic environment.^237^ Interestingly, HIF2α binds to the β-catenin–Tcf complex at a different site from HIF1α to recruit p300 and then enhances Wnt/β-catenin signaling.^210^ The synergistic actions of HIF2α and β-catenin increase the proliferation of renal cell carcinoma cells.^210^ Accordingly, utilizing Tanshinone IIA can inhibit CRC angiogenesis by means of interrupting HIF-1α/β-catenin/Tcf3/Lef1 signaling.^238,239^

Besides, HOXB13, SOX4, RUNX3, CDK8, TCTP, and Daxx participate in regulating Wnt/β-catenin signaling to target Tcf in numerous cancers. Specifically, HOXB13 expression was downregulated in colorectal and prostate cancer.^240,241^ And it inhibited the growth of cancers by reducing Tcf and c-myc protein levels.^240,241^ RUNX3 inhibited Wnt/β-catenin signaling by comprising of a ternary complex with β-catenin–Tcf and attenuates growth and progression of multiple cancers, especially in gastric cancer.^242,243^ SOX4 can increase β-catenin–Lef/Tcf transcriptional activity through upregulating Tcf4.^244,245^ CDK8, an oncogene in CRC, partly functions by co-activating β-catenin–Tcf complex.^246^ Thus, CDK8 may be a promising target for β-catenin-associated cancers.^246^ The translationally controlled tumor protein (TCTP) can enhance the transcription complex activity by increasing the ability of β-catenin–Tcf binding and then inducing the growth of glioma tumor.^247^ In summary, HOXB13 and RUNX3 are identified as suppressors of Wnt/β-catenin signaling by obstructing Tcf4 activity to inhibit tumorigenesis and cancer progression.^248,249^

#### Tnik

Traf2- and Nck-interacting kinase (Tnik) is one member of germinal center kinases (GCKs) that can activate the c-Jun N-terminal kinase pathway.^139^ Tnik is an essential component for Wnt/β-catenin signaling to maintain physiological cell homeostasis.^250,251^ Tnik can directly interact with β-catenin and Tcf to modulate Wnt/β-catenin signaling^250,251^ (Fig. 3). Therapeutically, Tnik is a crucial target for the treatment of CRC. In CRC cells, Tnik activates the transcriptional capability of Tcf4 through phosphorylation.^250,252^ It has been shown that the growth of CRC cells was strictly dependent on Tnik stimulation. Indeed, after knocking down Tnik, the growth of xenograft CRC cells was brought to stall.^251^ And patients with overexpression of Tnik were manifested with poor postsurgical outcomes.^253^ Over 80% of CRCs have mutations in Apc,^254^ which makes the only molecule downstream of Apc a therapeutic target.

## The crosstalk of Wnt/β-catenin signaling in cancers

### Wnt/β-catenin and Notch signaling

Notch signaling widely interacts with Wnt/β-catenin signaling in cell homeostasis and embryo development.^255^ Notch signaling also involves in the wingless development with Wnt/β-catenin signaling.^256,257^ Notch can directly inhibit Armadillo/β-catenin to enhance destruction complex activity.^256,257^ Besides, LNX2, a regulator of Notch, enhances the cell vitality in Wnt/β-catenin-associated CRC.^258^ In turn, β-catenin–Lef/Tcf can reciprocally activate Notch signaling by inducing the expression of Jagged1 and Dll1, which are the ligands of Notch signaling.^255,259^ In addition, β-catenin reduces Notch1 ubiquitination and increases the expression of Hes1 to interfere with Notch signaling transduction.^255,259^ The synergistic action of Wnt/β-catenin and Notch signalings promotes tumorigenesis and cancer progression. Apparently, the potential remedies of inhibiting this synergistic function can be beneficial in cancer therapies.^260–263^

### Wnt/β-catenin and Sonic hedgehog (Shh) signaling

The Sonic hedgehog signaling is another important interactor of β-catenin-dependent Wnt signaling.^9,264^ Smo, a receptor of Shh ligands,^265^ once being triggered via Shh, can activate Gli activities.^264–266^ Glis, including Gli1-3, are the ultimate effectors of Shh signaling as transcriptional regulators.^266^ Intriguingly, Gsk3β and CK1α can both phosphorylate and active Glis to reciprocally enhance Wnt/β-catenin signaling activity.^267,268^ The crosstalk of Shh-Wnt/β-catenin involves the relapse, invasion, and metastasis of certain cancers, so that the repression of this crosstalk may alleviate the progression, migration, and invasion of cancers.^269^ For instance, cyclopamine, as an inhibitor of Shh signaling, postpones the invasion of CRC by suppressing β-catenin–Tcf transcriptional activity.^270^

## Targeted therapies of Wnt/β-catenin in cancers

### Categories of pharmacology

Drugs targeting canonical Wnt signaling can be divided into two major subtypes, which are small-molecule inhibitor (SMI) and monoclonal antibody (mAb). SMIs usually refer to chemically synthesized compounds with a molecular weight of less than 1 kDa. In recent years, taking advantage of the in-depth learning of molecular and biological mechanisms in cancers, targeted drugs with fewer side effects plus better specificity than traditional chemo drugs have been developed. Theirs strengthens include but are not limited to (1) pronounced permeability into tissues and cells owing to the small-molecule weight; (2) larger volume of distribution; (3) diversified methods of drug delivery; (4) generally better oral tolerance and oral bioavailability after chemical modification. In contrast, mAbs cannot be taken orally, and are often administered by injection, which results in poor patient compliance. Even being directly injected, the distribution of mAbs in vivo is relatively limited. They cannot often easily reach therapeutic concentrations in some specialized tissues (such as the brain) as SMIs do. And as the inherent antigenicity of mAbs, they are likely to cause immune responses. mAbs are generally dominantly distributed in the kidneys, followed by the liver and spleen. In addition, they appear with non-linear pharmacokinetic characteristics, longer half-life, and smaller volume of distribution. Though mAbs can only act on extracellular targets, they have still achieved remarkable antitumor performances in comparison to SMIs, which is attributing to their different pharmacological mechanisms.

SMIs can bind to receptors with stronger affinity than the original ligands. On some occasions, they modify the structures of the receptors so that the ligands cannot be recognized. Moreover, certain SMIs may act as ATP analogs and bind to kinase sites in the cytoplasm, making the receptors unrecognizable.

The advantage of mAbs is not only to directly act on the extracellular and membrane-linked targets but also to activate the intrinsic internal immune system to indirectly exert antitumor effects. With respect to the direct functions, mAbs bind to receptors or ligands to block signal recognition or mediate internalization to reduce the density of receptors on the cell membrane surface.^271^ When acting indirectly, mAbs induce complement-dependent cytotoxicity (CDC), antibody-dependent cellular cytotoxicity (ADCC), or complement-dependent cell-mediated cytotoxicity (CDCC).^272,273^ Therefore, in in vivo experiments with a complete immune system, mAbs may show better antitumor effects than in in vitro experiments.^274^ In addition, mAbs can serve as the vehicle to achieve precision medicine, such as targeting radiogen to tumors.^275^

To sum up, in practical scenarios, mAbs are more competent for the treatment of hematological tumors than solid tumors as a consequence of their antigenicity and large particles.^274^ SMIs can target proteins even in the nucleus and penetrate the blood-brain barrier easily, thus preferentially suitable for treating solid tumors. Even if being humanized, mAbs are inevitable to arouse unintended immune reactions. Meanwhile, SMIs have poor antigenicity, but low specificity,^276^ which also brings a higher risk of side effects. Briefly, SMIs and mAbs exhibit diverse characteristics and various indications of cancers.

In addition to SMIs and mAbs, an under-developed category of Wnt/β-catenin-targeted therapy goes to peptides and peptide-associated modified drugs. Peptides have been put into use as drugs long ago, but obvious limitations delayed their clinical advancements. Peptidomimetic, a kind of peptide-associated modified drug, is much smaller than the parental molecule, yet having low antigenicity, good oral bioavailability, good permeability, and better diffusing to the target. Different from SMIs, their half-life is generally very short and has very rapid excretion. Although this feature reduces the risk of side effects, it contrarily causes difficulties to reach satisfactory concentration.^277^

## Promising preclinical targeted therapies

### SMIs targeting Porc

Porc inhibitors are well-acknowledged to block Wnt signaling with a low risk of off-target. Moreover, it was revealed that the exhaustion of canonical Wnt ligands prevented cancer cell proliferation but induced differentiation. Therefore, inhibiting Porc may be a mild therapy that could prevent tumor growth rather than directly causing lethal effects. It has been previously described that Znrf3/Rnf43 are important negative regulators of Wnt signaling. The LoF mutation of Znrf3/Rnf43 was reported to be oncogenic, and Porc inhibitors had shown a remarkable potency in this aspect.^27,124,278^ Unfortunately, the absence of Apc is likely to induce a Wnt ligand-independent signaling, so tumors with Apc LoF mutations may be resistant to Porc inhibitors.^279^ And Picco et al. reported that in a CRC cell line (VACO6) with RSPO3 fusion, long-term exposure (3 months) of a Porc inhibitor (LGK974) with incremental doses can induce drug resistance. This kind of drug resistance is accomplished by LoF of Axin1 in the VACO6.^160^

Contemporarily, there are a growing number of reports showing the side effects of Porc inhibitors, among which bone loss is the most frequent outcome.^280,281^ Two studies indicated that LGK974, WNT-C5, or ETC159 can lead to bone loss by diminished osteogenesis and increased osteolysis. These bone loss phenotypes were reported to even appear under the condition of effective dosages of Porc inhibitors.^282–284^ Hopefully, this side effect can be relieved by co-administrating diphosphonate (alendronate, zoledronic acid for instance).^280^ And other adverse effects may be solved by lower doses through the incorporation of other antitumor drugs.

#### LGK974

Also known as WNT974, LGK974 is a potent SMI targeting Porc. A preclinical study showed the effective performance of LGK974 among variable neoplasm models with good oral tolerance.^282^ Even this study systematically examined many organs such as the intestine, stomach, and skin, but unfortunately giving an ignorance to bone tissue. Besides, it was discovered that all human head and neck squamous cell carcinoma (HNSCC) cell lines with Notch nonsense mutation were more sensitive to LGK974.^282^ Inspired by this, a team signed up for a clinical trial attempting to use LGK974 to treat HNSCC patients with Notch LoF mutation^285^ (Table 2). But this project was abandoned as found retrieval. Another study showed the incredible use of LGK974 in Rnf43 nonsense mutation cell lines in pancreatic ductal adenocarcinoma (PDAC).^124^ Both of the two clinical studies illustrated a delayed effect of proliferation inhibition by LGK974. Specifically, after a single administration, there still remains some β-catenin in the cytoplasm to initiate the downstream transcriptional activities. LGK974 also preliminarily demonstrated a reliable tumor-suppressive effect in vitro on neuroendocrine tumor and ovarian cancer.^286,287^

**Table 2:** The summary of clinical trials estimating the agents targeting canonical Wnt signaling in cancers

Target/role	Name	Clinical trials (Phase)	Condition
PORCN/inhibitor	LGK974	NCT01351103^289^ (P1); NCT02278133^288^ (P1/2); NCT02649530^285^ (P2)	Pancreatic cancer, melanoma, BC, cervix cancer, esophageal cancer, CRC, HNSCC
	ETC-159	NCT02521844^290^ (P1b); Teneggi et al.	CRC, OC, endometrial cancer
	CGX-1321	NCT02675946^294^ (P1)	Gastrointestinal tumor
FZD-1, 2, 5, 7, 8/mAb	OMP-18R5	NCT01345201^418^ (P1b); NCT02005315^419^ (P1b); NCT01957007^420^ (P1b); NCT01973309^419^ (P1b); Diamond et al.; Davis et al.	Pancreatic cancer, NSCLC, HER2^-^ BC
Truncated FZD8 fused to IgG1 Fc	OMP-54F28	NCT01608867^421^ (P1); NCT02069145^422^ (P1b); NCT02092363^423^ (P1b); NCT02050178^424^ (P1b); Dotan et al.; Moore et al.; Jimeno et al.	Sarcoma, basal cell cancer, PC, HCC, OC, metastatic pancreatic cancer
RSPO3/mAb	OMP-131R10	Bendell et al.^314^	CRC, ovarian cancer
WNT5a/peptide mimic	Foxy-5	NCT02020291^425^ (P1); NCT02655952^426^ (P1); NCT03883802^427^ (P2); EUCTR2018-003074-27-ES (P2)	BC, PC, CRC
Dkk1/mAb	DKN-01	NCT03645980^318^ (P1/2); NCT01711671^428^ (P1); NCT01457417^429^ (P1); Goyal et al.^430^ EUCTR2018-004138-13-GB (P2); NCT04057365^431^ (P2); NCT03645980^432^ (P1/2)	HCC, esophagealgastric cancer, MM, NSCLC, biliary tract cancer
ROR1/mAb	UC-961	NCT02222688^433^ (P1); NCT03088878^434^ (P1b/2); NCT02776917^435^ (P1b); Choi et al.^436^	CLL, small lymphocytic lymphoma, BC, MCL
FZD-7	TcdB-FBD	ChiCTR1800018069	BC
CK1δ, ε/inhibitor	Umbralisib	NCT04163718^437^ (P2); NCT03776864^438^ (P2); NCT04692155^439^ (P1/2)	CLL, HL, MCL
GSK3/inhibitor	LY2090314	NCT01214603^440^ (P2); NCT01287520^441^ (P1); Rizzieri et al.^442^; Gray et al.^366^	AML
CBP/βcat inhibitor	PRI-724/ ICG-001	NCT01302405^443^ (P1); NCT01764477^444^ (P1b); NCT02413853^445^ (P2);	Pancreatic cancer, CRC
TCF/βcat	BC2059	NCT03459469^446^ (P1)	Desmoid tumor

A study using uncovered similar impairment of bone mass in two different doses of LGK974 in mice (3 and 6 mg/kg/d for 7 days).^281^ Another study expanded the dose range (from 1to 30 mg/kg/d) and showed an all ranges covered bone loss after continuous treatment for 4 weeks.^280^ What’s worrying is that the dosage of 3 mg/kg/d for mice is necessary to reach a significant tumor repression.^282^ And the exposure to a high dose of LGK974 (20 mg/kg/d) results in prominent intestinal toxicity.^282^ Fortunately, as the sustaining treatment of LKG974 is not required for tumor treatment, the practical clinical dosage may not bring so many side effects. Notably, the clinical dosage of LGK974 is still in exploration without explicit results posted.^288,289^

#### ETC159

ETC159, originally named ETC-1922159, is a potent orally available SMI for Porc. Madan et al. identified its high efficiency in CRC with Rspo translocations. This study also inspected intestinal tissues, but still neglected bones.^283^ ETC159 can perform effectively synergistic effects with PI3k/mTor inhibitor GDC-0941 in PDAC with Rnf43 LoF mutation.^278^ Based on the outstanding behavior of ETC159 in a preclinical experiment, another phase II clinical trial targeting advanced solid tumors has been carried out. Interestingly, part B of this trial evaluates the combination usage of ETC159 and pembrolizumab. But no results have been posted up to date^290^ (Tabl. 2).

Bone loss has been observed after 4 weeks of administration in a dosage of 3–30 mg/kg/d. The research team adjusts administration frequency from *qd* to *qod* to validate the hypothesis of whether the bone loss could be attenuated after a full metabolic cycle of ETC159. Results showed that there were no significant differences in several estimated outcomes of bone between treated daily and treated every other day.^280^ By the way, ETC131, a similar compound of ETC159, is only used for in vitro assays as a result of its inferior oral bioavailability.^283^

#### CGX1321

CGX1321 is a novel Porc inhibitor, yet only a few studies have researched into this small molecule. For experimental analysis, it has been discovered for a good performance in CRC with fused Rspo.^291^ Further in-depth studies respectively included the combined usage of CGX1321 and immune checkpoint blockade therapy to treat ovarian cancer (but the results were unsatisfactory),^292^ and liposome encapsulation as a vehicle to deliver CGX1321 to treat cancer stem cells (with expected results).^293^

As for the clinical trial of CGX1321, it was solely used in solid tumors including gastrointestinal tumors (Table 2). And the combination of CGX1321 with pembrolizumab in CRCs was constructed to evaluate the safety.^294^ Yet no results have been posted currently.

#### WNT-C59

This drug is not well-studied when compared with LGK974 or ETC159, but it exclusively targets mammalian Porc. It owns good oral tolerance and efficacy in the MMTV-Wnt1 mouse mammary cancer model,^295^ the Znrf3/Rnf43^−/−^ mouse CRC model.^284^ And WNT-C59 satisfactorily prevented tumor growth in mice xenografted with SUNE1 or HNE1 (two nasopharyngeal carcinoma cell lines).^296^ In addition, WNT-C59 can reverse the resistance of trichostatin A in human pancreatic cells.^297^ However, when administrated at 10 mg/kg/d for 7 or 21 days, bone loss was observed in mice.^281^ This should be taken into consideration as the potential side effect of WNT-C59 in cancer therapies.

#### GNF-1331/GNF-6231

GNF-1331 is a precursor drug, and the optimization of it brings on the discoveries of LGK974 and GNF-6231.^298^ Currently, GNF-6231 is still a brand-new molecule without clinical studies. But it worked well in MMTV-Wnt1 patient-derived xenograft (PDX) mice model, by reducing Axin2 expression.^298^

#### IWP

Relying on the screening approach of the phenotype of inhibition of Wnt production, this kind of molecule was named as “inhibitors of Wnt production (IWP)”. In this study, the research group then verified the exclusive activity of inhibiting Wnt-related Porc function by using IWPs.^299^ IWP has three family members, IWP-1, −2, and -L6, all of which oppress Wnt/β-catenin signaling by competing for the active sites of Porc with Wntless.^299^ IWP-2 and IWP-L6 had poor metabolic stability in mice, but at least IWP-L6 showed relatively better stability in human.^300^ IWPs may synergist with PRI-724 to induce apoptosis in HNSCC through inhibition of both Porc and CBP/β-catenin.^301^ However, as IWPs have a similar structure to certain CK1 inhibitors, much research have proposed the effects on CK1 isoform suppression of IWPs.^302,303^ IWP-2 and IWP-4 inhibited the proliferation of various cancer cells via antagonizing Porc and CK1α.^303^ The growth of gastric cancer cells was restrained via the IWPs-mediated downregulation of Wnt/β-catenin signaling.^304^ A similar situation occurred in HNSCC cells as well.^301^

### mAbs targeting Wnt receptors and co-receptors

mAbs are remarkably targeted drugs especially for blocking extracellular or membrane-linked proteins. They are characterized by high affinity and low off-target accidents but have longer plasma half-life and lessened clearance rate.^305^

#### OMP-18R5/OMP-54F28

Also known as vanticumab, OMP-18R5 is a monoclonal antibody that targets human Fzd1, 2, 5, 7, and 8. And it was reported to inhibit the growth of gastric adenomas in mice models, either with or without Apc LoF mutation.^84^ OMP-54F28, namely ipafricept, is the chimera of truncated Fzd8 and IgG1 Fc region.^306^ Ipafricept shares many features with vanticumab. A study has proved the high antitumor effect of ipafricept in MMTV-Wnt1 cancer models. Moreover, the synergistic utilization of ipafricept gemcitabine demonstrated inhibitory function in a pancreatic PDX model, and significantly reduced the cancer stem cells (CSC) frequency. This combined strategy showed superior capabilities of tumor arrest over the sole usage of ipafricept either or gemcitabine.^307^

Encouragingly, either vanticumab or ipafricept exhibited impressive synergistic therapeutic effects with taxanes (paclitaxel, docetaxel, and cabazitaxel). Fischer et al. discovered that there existed a population of taxane-resistant cancer cells after using paclitaxel. And the successional administration of ipafricept/vanticumab prior to paclitaxel can overcome the drug-resistant effect via a sole administration of paclitaxel. Mechanically, this combined usage of ipafricept/vanticumab and paclitaxel strengthened the mitotic catastrophe.^308^

The adverse effects of OMPs are similar to Porc inhibitors, but OMPs showed worse impairment of bone mass. As for ipafricept, in a clinical trial in patients with advanced solid tumors, fragility fractures were reported in two participants at 20 mg/kg q3w.^309^ Another trial recruited patients inflicted with recurrent ovarian cancer, in which a patient experienced a pelvic fracture at 5 mg/kg q3w.^310^ Studies pointed out that vanticumab had a more severe side effect on bone than ipafricept. In detail, in a clinical trial with metastatic pancreatic cancer, two participants experienced fragility fractures at 7 mg/kg q2w.^311^ Additionally, in a trial attempted to treat advanced or metastatic HER2^-^ breast cancer, vanticumab led to fragility fractures in 3 patients, including three Grade 2 events and one Grade 3 events of fracture ranking, at the regimen of 7, 14 mg/kg q2w and 8 mg/kg q4w. Surprisingly, this unintended outcome appeared under the critical supervision of bone metabolism outcomes and bone anabolic remedy of diphosphonate treatment. At last, a total of 6 patients experienced fragility fractures, leading to the abolishment of this trial.^312^ All those fracture events mentioned above were not reported as dose-limited toxicity, because they took place after the 1st 28d-window-phase. Whereas, still no studies could reach the maximum administered dose (MAD) in consideration of bone safety. Overall, though preclinical experiments exhibited a good performance of OMPs, these trials revealed a crisis of applying ipafricept/vanticumab in clinical (Table 2).

#### OMP-131R10

Also named as rosmantuzumab, OMP-131R10 is a humanized mAb targeting Rspo3. Some malignant hematopoiesis cancers have aberrant Wnt/β-catenin signaling independent to Wnt ligands, due to the redundancy of Rspos in triggering Wnt/β-catenin signaling. Based on this, Salik et al. demonstrated that rosmantuzumab can impair the self-renewal and differentiation of acute myeloid leukemia cells in the PDX model, meanwhile free from influencing normal hematopoietic stem cells.^313^ For the clinical trial, a phase 1a/b trial about rosmantuzumab in advanced solid tumors and previously treated metastatic CRC is still going on^314^ (Table 2).

#### F2.A

F2.A is a newly developed antibody that targets 6 of the 10 human Fzds (Fzd1/2/4/5/7/8). The developer synthesized this compound by firstly identification of anti-Fzd antibodies (F2) with a specific profile matching to OMP-18R5 and secondly using combinatorial antibody engineering to find a variant F2.A with specificity to bounding Fzd4. According to their study, F2.A could selectively bound to Fzd4 without competition with Norrin. Moreover, F2.A had a much better potency when treating Rnf43 mutated PDAC when compared to OMP-18R5 and OMP-54F28.^315^

#### DKN-01

DKN-01 is a humanized IgG4 mAb that can bound and block the activity of Dkk1. Physiologically, Wnt/β-catenin signaling activates the transcription of Dkk1, which in turn bounds LRP5/6 and block the recognition of Wnt ligands, forming a negative feedback loop. However, some tumors featured with overwhelmed Wnt/β-catenin signaling are insensitive to Dkk1. In contrast, the superfluous Dkk1 can promote tumor cell proliferation. Though the specific mechanisms are unknown, it was hypothesized that Dkk1 helped tumors escape from immune supervision.^316^ Indeed, it was reported that an intact immune system was needed for DKN-01 function in a murine model.^317^ Most clinical trials are researching on DKN-01, most of which focused on the digestive system such as the gastroesophageal, intestine, liver, and biliary tract cancers, and the rest focused on NSCLC, gynecologic malignancies, and multiple myeloma. DKN-01 showed satisfactory tolerance in all clinical trials. Intriguingly, some trials reported a better effect of DKN-01 in Dkk1-overexpressed patients^318^ (Table 2).

#### UC-961

UC-961, also called cirmtuzumab, is a first-in-class mAb that targets ROR1. ROR1 is often highly expressed in chronic leukemia lymphoma (CLL) cells. As normal B lymphocytes do not express ROR1, UC-961 has precise and good effects in treating CLL.^319^

#### OTSA-101

Fzd10 was found ubiquitously upregulated in synovial sarcoma (SS), but scarcely detectable in normal tissues except the placenta.^101^ A group of radioimmunoconjugate humanized antibody OTSA-101 was designed to target Fzd10, whose derivations include ^211^At-OTSA-101, ^111^In-OTSA-101, and ^90^Y-OTSA-101. Among them, ^111^In-OTSA-101 is routinely used as a diagnostic tool. Whereas OTSA-101 only showed a weak antagonistic activity on the growth of SS cells. Afterall, OTSA-101 merely became a putative carrier for targeted SS radiotherapy. In preclinical experiments, a large number of cell death occurred in the PDX murine model on the first day after injection of ^211^At-OTSA-101. All mice in this interventional group survived, and the tumor volume was greatly diminished. However, ^211^At-OTSA-101 was easily accumulated in the stomach and its uptake rate of tumor cells was not as good as ^111^In-OTSA-101. But the inhibitory effect of ^211^At-OTSA-101 was much better than ^90^Y-OTSA-101^281^. Furthermore, ^90^Y-OTSA-101 showed obvious bone marrow suppression and significant hematotoxicity.^320^

### Peptide mimetics

#### Foxy-5

Sponsored and developed by WntResearch (https://www.wntresearch.com), foxy-5 is a mimic of WNT5A. Preclinical studies showed that a low level of WNT5A was correlated with a more metastatic or advanced outcome in breast and prostate cancer. In accordance, foxy-5 could prevent metastasis to some extent.^321,322^ At present, WntResearch has supported five clinical trials registered on NIH and EUCTR, yet with no results posted (Table 2).

#### CWP232291

CWP232291, abbreviated as CWP291, can decline the transcriptional effect of canonical Wnt signaling. CWP291 was reported to suppress the growth of castration-resistant prostate cancer through the degradation of β-catenin via apoptosis-induced ER stress.^323^ In fact, CWP232204 is the active form of CWP291 in serum. Currently, CWP291 was put forward in clinical trials of treating acute myeloid leukemia (AML) and refractory myeloma without results updated (Table 2).

### SMIs targeting cytoplasmic proteins

#### Tnks inhibitors

Tankyrase is a poly-ADP polymerase (PARP), which can make Axin1/2 poly-ADP ribosylation, leading to the ubiquitination and degradation of the latter. Therefore, the silence of Tnks will result in the inhibition of the Wnt/β-catenin signaling. According to the binding sites, Tnks inhibitors can be divided into two categories: targeted nicotinamide subsites and adenosine subsites. The nicotinamide domain is ubiquitous in the PARP enzymes, and its specific site inhibitor is XAV939. For the adenosine domain, it is unique to Tnks, and many Tnks inhibitors (G007-LK, NVP-Tnks656, JW55/74, and IWR) target this site.^324^

Having been developed for a long time, Tnks inhibitors have shown outstanding tumor-suppressive effects in preclinical experiments, but none of them have entered into clinical trials. Of note, they were reported to possibly cause bone loss. Inhibition of Tnks (using either XAV939, IWR-1, or G007-LK) in murine models led to the accumulation of a substrate, SH3 domain-binding protein 2 (SH3BP2), which subsequently enhanced the Rankl-mediated osteoclast formation.^325,326^ In the human genome, the gain of function (GoF) of SH3BP2 promoted osteoclastogenesis so which led to bone loss.^327^ Although SH3BP2 can also promote the differentiation and maturation of osteoblasts,^328^ Tnks inhibitors dominantly showed an osteoclastogenesis effect in vivo, because the concentration needed for stimulating osteoblast is about 10 times higher than that of osteoclasts.^326^ Not only Tnks, but the family of PARP plays an important role in bone homeostasis. Though theoretically, pan-PARP inhibitors are presumed to have a greater influence on bone, there is currently no convincing evidence to prove their regulations of bone mass.

In addition, Tanaka et al. found that CRC cells (whether established cell lines or patient-derived cells) with Apc-truncated mutations responded well to Tnks inhibitors (XAV939, IWR-1, and G007-LK), especially the mutations with all the 20-amino-acid repeats (the β-catenin binding sites of Apc) obliterated. Conversely, the longer Apc mutation may lead to resistance to Tnks inhibitors. These results suggested an underlying therapeutic value of Tnks for dealing with truncated Apc mutant cancers.^329^

XAV939 was identified as an inhibitory factor of Wnt/β-catenin signaling, originally in CRC cell lines. This SMI stabilized Axin to increase β-catenin degradation by suppressing Tnks1/2.^171^ It has been investigated that beside from CRC, XAV939 can constrain certain cancers by inhibiting the Wnt/β-catenin signaling. The combined utilization of low-dose paclitaxel and XAV939 inhibited breast cancer metastasis and the growth of triple-negative breast cancer. This mechanism was contributed to inhibiting Wnt/β-catenin signaling to enhance cancer cell apoptosis and attenuate EMT and angiogenesis.^330^ In gastric cancer, XAV939 can inhibit the invasion and metastasis of cancer cells.^331^ XAV939 and RNAi-Tnks1 inhibited the stemness and migration of cancer stem cells and accelerated cell apoptosis in neuroblastoma by attenuating the abnormal status of Wnt/β-catenin signaling.^332,333^ XAV939 and IWR, the inhibitors of Tnks, repressed the growth of lung cancer and reduced tumorigenesis. Experimental data proved the inhibitory effect of XAV939 and IWR on cancer cell growth in murine lung cancer models.^334^ XAV939 enhanced the ability of CD4^+^ lymphocytes in biochemically recurrent prostate cancer cell lines, LNCaP and PC-3.^335^

JW67 and JW74, two compounds as inhibitory molecules of Wnt/β-catenin signaling, can inhibit the growth of CRC by Axin2 accumulation and β-catenin degradation.^336^ JW74 and JW55 are Tnks inhibitors that bind to the lower part of donor NAD^+^ cleft, instead of mimicking nicotinamide. JW55 also worked well in the murine PDX CRC model with Apc mutation.^337^ G007-LK is an analog of JW74, and G244-LM is analogous to XAV939.^171,336^ Compound G007-LK and G244-LM can damage the proliferation, colony formation, and growth of CRC cells via activating Axins to suppress Wnt/β-catenin signaling.^338^ As an adjuvant, G007-LK can also enhance the sensitivity of glioma stem cells to a chemo drug temozolomide and CRC cells to PI3K/EGFR inhibitors.^339,340^

As mentioned above, IWR compounds were defined based on their anti-Wnt pathway activities in a phenotypic screening assay.^299^ IWRs, including five molecules, target Wnt-dependent cancers by enhancing Axin capability to suppress Wnt/β-catenin signaling.^299,341^ The inhibition of IWRs has been identified in various cancers like osteosarcoma, colorectal, breast, lung, and hepatocellular cancers.^169,299,342–345^ IWR-1 served as a good adjuvant that reversed the resistance of osteosarcoma to doxorubicin, and in vivo inhibited the growth of subcutaneous PDX osteosarcoma.^342^ Moreover, IWR-1 could inhibit the EMT of CRC by inhibiting Wnt/β-catenin transduction.^343^

In the following part, we are going to discuss several agents, NVP-TNKS656, AZ1366, RK-287107, and HLY78, that are Tnks inhibitors with very limited preclinical reports. For instance, NVP-TNKS656 increased the sensitivity of CRC cells to PI3K/AKT inhibitor in vivo, but the increment effect could be reversed by high FOXO3A.^346^ And NVP-TNKS656 also inhibited the metastatic and invasive EMT hallmarks of hepatoma carcinoma cells.^347^ AZ1366 is a novel inhibitor of Tnks to constrain NSCLC growth. EGFR-driven NSCLC was suppressed by the synergistic function of AZ1366 and EGFR inhibitors.^348^ AZ1366 could erase the insensitivity of CRC cells to irinotecan,^349^ and it coordinated with EGFR inhibitor to control the growth of Wnt-responsive lung cancers in the murine models.^348^ RK-287107 was claimed to inhibit Tnks1/2 four or eight times more than G007-LK. It could down-regulate the Wnt/β-catenin signaling in Apc-truncated CRC cells, but had little effects in wild-type cancer cells.^350^ An SMI from the synthetic chemical library of lycorine derivatives, 4-ethyl-5-methyl-5,6-dihydro-[1,3]dioxolo[4,5-j]phenanthridine (HLY78), activated Wnt/β-catenin signaling by targeting the DIX domain of Axin and enhanced the effect of Axin-Lrp5/6.^351^

#### CK1 inhibitors

The family of CK1 proteins has several isoforms, of which CK1α/δ/ε are involved in the canonical Wnt signaling, but their roles are distinguished. CK1α serves as a component of the destructive complex and phosphorylates β-catenin. Instead, CK1δ/ε phosphorylates Dvl leading to the stabilization of β-catenin. And CK1δ and CK1ε are also moonlighting proteins that control the circadian clock. IC261 is a selective, ATP-competitive CK1 inhibitor that has shown its high efficiency in handling CRC and glioblastoma cells.^352,353^ Not only that, IC261 has alternatively biological effects that may lead to the death of cancer cells independent of inhibiting the canonical Wnt pathway.^352,354^ In an in vitro study, IC261 induced centrosome fragmentation during mitosis independent of CK1δ.^355^ Other CK1 inhibitors include PF670 and PF480 that do not kill cancer cells.

Also known as umbralisib, TGR-1202 is a dual kinase inhibitor that targets both PI3Kδ and CK1ε. The non-canonical Wnt pathway has been demonstrated to play an important role in CLL with CK1δ/ε overexpression.^356^ TGR-1202 is exclusively used in hematological malignancies and is currently recruited in clinical trials for treating CLL, Hodgkin’s lymphoma, mantle cell lymphoma, and so on (Table 2).

Pyrvinium, known as an antiparasitic drug, has been approved by FDA as an orphan drug for FAP because of its exclusive CK1α agitation capability. Earlier experiments have shown that pyrvinium inhibited the proliferation of HCT116 and SW480 cell lines by selectively activating CK1α and inhibiting canonical Wnt signaling.^194^ This activation may be achieved by allosteric regulation, thereby improving the catalytic ability of CK1 without affecting the binding of substrates.^357^ Although pyrvinium also showed inhibitory ability in other Wnt/β-catenin signaling-driven tumor cell lines (SUM-149/SUM-159,^358^ and A2278^359^), its bioavailability was low in tissues other than the intestine.^360^ In addition, pyrvinium also stimulated CK1γ, leading to Wnt signaling suppression. However, some scholars indicated pyrvinium pamoate lacked the efficacy on CK1, instead, it downregulated AKT by an undiscovered mechanism to activate Gsk3β.^361^

#### Gsk3 inhibitors

Ambiguously serving as carcinogenic or cancer suppressor,^362^ Gsk3 lies downstream of diverse signaling pathways, including the Wnt/β-catenin signaling pathway. Therefore, targeting Gsk3 has very restricted specificity to produce unexpected off-targets. Thus, this review will not thoroughly discuss Gsk3 inhibitors due to their low specificity of Wnt/β-catenin signaling. Therefore, here only lists a typical Gsk3 inhibitor that has shown good performance in inhibiting Wnt/β-catenin signaling.

LY2090314 is a potent Gsk3α/β inhibitor that has demonstrated good cooperation with platinum agents in vitro in the treatment of melanoma,^363^ AR-V7^+^ prostate cancer,^364^ and neuroblastoma.^365^ But in a phase I clinical trial, the combination of LY2090314 and pemetrexed/carboplatin showed eleven DLTs in ten enrolled patients^366^ (Table 2). Other Gsk3 inhibitors like ABC1183 and CHIR-99021 are currently under exploring.

#### Dvl inhibitors

Though serving as a critical conductor in the canonical Wnt pathway, only few drugs and studies targeting Dvl are available. Till now, targeting Dvl has already shown a notable potentiality for tumor treatment. For instance, Dvl2 was overexpressed in HCC and reported to link with poor prognosis.^367^ In comparison to normal adult bronchial/alveolar epithelial and peripheral blood mononuclear cells, Dvl1-3 were found to be exclusively expressed in NSCLC and CLL cells, respectively.^368,369^ Most drugs targeting Dvls currently are developed to selectively inhibit PDZ-Fzd interaction. Such as FJ9, it was significantly reported to cause apoptosis in melanoma and NSCLC cell lines.^370^ 3289-8625, another Dvl inhibitor, suppressed the growth of PC-3 cells.^371^ Taken together, the druggability of Dvls remains largely unknown. Urgent studies are demanded to unearth more applicable chances for drugs targeting Dvls in the future.

### Agents targeting protein–protein interaction (PPI) in the nucleus

**Fig. 4:**
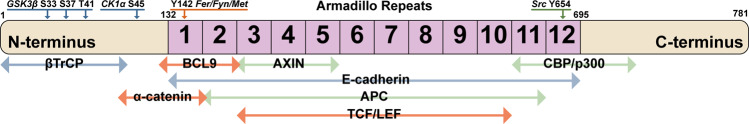
The structural illustration of human β-catenin protein. In this figure we mainly demonstrated the important PPI binding domains and phosphorylation sites of β-catenin. This image was modified from a published research.^372^

Nuclear localization of β-catenin will initiate downstream gene expressions. This process involves the formation of a key transcription complex, β-catenin–Lef/Tcf. β-catenin-dependent transcriptional regulation is also modulated by the phosphorylation protein Tnik, and various transcription co-factors like CBP, BCL9, CREB, BRG1, etc. These proteins listed above are all potential therapeutic targets of Wnt/β-catenin signaling. Despite the most core position of β-catenin in canonical Wnt signaling, pityingly, among all previously screened SMIs, few can directly bind to β-catenin. This is because, unlike most enzymes or receptors with identifiable binding pockets, the PPI surface of β-catenin is relatively large and flat for small molecules. And the Tcf-binding domain within β-catenin is overlapped with many other proteins, making it difficult to specifically interfere with Tcf/Lef^372^ (Fig. 4). Moreover, among the limited SMIs (CWP232228 for example) that can directly bind to β-catenin, there lacks evidence of Wnt signaling inhibitory in vivo. In contrast, targeting Tnik showed more stable inhibition of canonical Wnt signaling.

#### Tnik inhibitors

As mentioned above, Tnik is a critical therapeutic target of CRC. Among the Tnik inhibitors, the most commonly used are aminothiazole-based. Masuda et al. discovered a series of SMIs called NCB, classified as ATP-competitive inhibitors. After a high-throughput screening of the small-molecule compound library, they firstly found NCB-0001 (with a moderate inhibitory effect on canonical Wnt signaling in HEK293 cells), which led to the discovery of N5355 following the structural optimization.^252^ N5355 significantly reduced the expression of Wnt/β-catenin-dependent genes, such as Axin2 and cMYC, in HCT-116 cell line.^373^ What’s worth noting, N5355 did not affect the vitality of canonical Wnt signaling independent cell lines, HELA and HEL299.^252^ This research group subsequently discovered NCB-0005 (or called KY-05009), which markedly inhibited the activation of Wnt/β-catenin signaling mediated by TGF-β1 in A549 cells.^373^ Also, the NCB-0005 cooperated with the RTK inhibitor dovitinib to impede the growth of multiple myeloma cells.^374^ Their latest discovery was NCB-0846, which inhibited the growth in a variety of patient-derived CRC xenografted tumors,^375^ and abrogated the EMT of DLD-1, HCT-116, and A549 cell lines.^376,377^

As ATP-competitive inhibitors, NCB series drugs may also have inhibitory effects on other kinases. Therefore, it should be concerned that the clinical effects of NCB drugs may not be limited to target canonical Wnt signaling. In addition to CRC, inhibitory effects of NCB drugs have also been reported in other tumors.^378,379^

#### Inhibitors targeting β-catenin–-Lef/Tcf complex

Such inhibitors block the PPI between β-catenin and Tcf/Lef. However, the Tcf/Lef recognition domain on β-catenin highly overlaps with that of Axin, Apc, E-cadherin, and other proteins, which may bring potential adverse effects derived from off-targets of canonical Wnt signaling.

In this part, we will mainly discuss the β-catenin–Lef/Tcf complex inhibitors as following: CWP232228, LF3, BC2059, PKF115-584, PKF222-815, and CGP049090. CWP232228 is such an inhibitor, which was reported to induce apoptosis and cell cycle arrest in HCT116 cells,^380^ also to show effects on liver and breast CSCs.^381,382^ LF3, a 4-thioureido-benzenesulfonamide derivative, reduced tumor growth and induced differentiation in a CRC xenograft murine model.^383^ Moreover, BC2059, an anthraquinone oxime-analog repressing Lef1/Tcf4 activity, was elucidated to induce the apoptosis of HL-60, HEL, and K562 significantly.^384,385^ BC2059 is currently testified in a phase I clinical trial of desmoid tumor (Table 2). Other drugs of β-catenin–Lef/Tcf complex inhibitors, including three fungal derivatives, PKF115-584, PKF222-815, and CGP049090, have shown prominent inhibitory effects towards CLL/AML (PKF115-584 and CGP049090) and HCC (all three agents) cells, through inducing apoptosis.^386–388^ Still, some β-catenin–Tcf/Lef inhibitors need more preclinical experiments to verify their practicabilities, such as PNU74654 and 2,4-diamino-quinazoline derivatives.

#### Transcription co-factor inhibitors

For less superposition and narrower PPI surface in the recognition domain, inhibitors targeting the terminal domains (unstructured or intrinsically disordered protein regions) are putative to be more specific and druggable. These regions involve the binding of transcriptional co-factors like BCL9/B9C and CBP. Indeed, the SMI 40-fluoro-*N*-phenyl-[1,10-biphenyl]−3-carboxamide and its derivatives showed a much greater affinity toward β-catenin/BCL9 over β-catenin/E-cadherin.^389^

For targeting CBP, PRI-724, and ICG-001, a pair of enantiomers, are potent inhibitors of canonical Wnt signaling that antagonize the binding of β-catenin to CBP but not affecting the viability of p300 meanwhile. They have similar effects and sometimes can be used interchangeably. Their preclinical efficiency has been demonstrated in a variable of cancer cells such as HNSCC,^301^ hepatoma carcinoma,^390^ and neuroendocrine tumor cells.^286^ A clinical trial of cancer research is still engaging but without results posted (Table 2, two of the three trials were terminated). However, they seemed to be more applicable in antifibrosis than in antitumor.^391,392^

BCL9 provides an excellent opportunity for targeting Wnt signaling therapy. In normal cells, BCL9 expression is almost undetectable,^393^ but it reigns a set of EMT-regulated Wnt target genes in cancer cells.^394^ And after knocking out Bcl9 and B9l in mice, no obvious harmful phenotypes were found, indicating that these genes are of little importance for mammals to balance Wnt signaling in normal tissues.^394^ Based on this, Takada et al. developed the stapled peptide stabilized α helix of BCL9 (SAH-BCL9), which can snatch β-catenin from the endogenous Bcl9/Tcf/β-catenin complex with greater affinity. SAH-BCL9 showed a significant antitumor effect in both colo320 in vitro and intraperitoneally injected murine model in vivo.^395^

#### Other drugs may target Wnt/β-catenin signaling

Apart from the synthetic compounds, in recent years more and more natural products have been revealed to impact on repressing Wnt/β-catenin signaling.^396–399^ The most studied may be resveratrol, a phytoalexin produced when plants are inflicted with injuries or pathogenic attacks. Resveratrol has been enrolled into two phase I clinical trials, investigating its value in the dietary supplement in CRC prophylaxis.^400,401^ Moreover, silibinin, an extract from the milk thistle seeds, also showed its potentiality in preventing tumorigenesis in a preclinical model.^402,403^

In addition to natural products, many FDA-approved drugs have expanded their indications to crosstalk with Wnt/β-catenin signaling. Just as mentioned above, as an antiparasitic agent pyrvinium also inhibited Wnt/β-catenin pathway via activating CK1α, or Gsk3β.^194,361^ Other old drugs like niclosamide to inhibit canonical Wnt signaling by promoting Fzd1 endocytosis,^404^ celecoxib to induce the degradation of Tcf7,^405^ and salinomycin to target LRP6.^406^ Due to the functions of these old drugs for targeting Wnt/β-catenin signaling are still limitedly known, we will not continue to discuss these drugs. For more details, please find in Table 3.

**Table 3:** The summary of up-to-date drugs that have implications on Wnt/β-catenin signaling in cancers

Name	Original use	Possible mechanisms on Wnt signal	Condition
Natural products			
Resveratrol	Dietary supplement	Stimulating proteasomal of TCF4	CRC (HCT116, SW480, HT-29, LoVo, Caco-2)^447^
		Unknown	Glioma (GBM2, GBM7, G144, G179, G166, GliNS2, GBM04)^448^
		Insufficient βact-oriented siRNA	SCC (Colo16)^449^
Quercetin		Inhibiting βcat unclear translocation	Teratocarcinoma (NT2/D1)^450^
		Unknown	PC-3^451^
		Inhibiting Tcf transcription activity	CRC (SW480)^452^
Isoquercitrin		Inhibiting βcat unclear translocation	CRC (SW480, DLD-1, HCT116)^453^
Curcumin		Inducing caspase-3-mediated β-catenin degradation	CRC (HCT-116)^454^
		Reducing expression of βcat and Dvl	BC (MCF-7, MDA-MB-231)^455^
		Reducing nuclear βcat level	OS (U2OS, SaOS-2, HOS)^456^
		Reducing Tcf4/CBP/p300 levels	PC (22rv1, DU-145)^457^
		Inhibiting GPC3/TPA-induced Wnt signaling activation	HCC (HepG2, Hep3B)^458,459^
		Reducing expression of βcat	NSCLC (A549)^460^
Silibinin (Silybin)	Hepatoprotectant	Inhibiting Lrp6 promoter activity	PC (PC-3, DU-145)^461^; BC (MDA-MB-231, T-47D)^461^
		Inhibiting Tcf4 transcription activity	CRC (SW480, HCT116)^462^
		Unknown	Lung cancer (PC9, A549)^463^
Rottlerin	PKC inhibitor	Promoting Lrp6 degradation	PC (PC-3, DU-145)^398^; BC (MDA-MB-231, T-47D)^398^
		Reducing expression of Lrp6 and βcat	ACC (NCI-H295R, SW-13)^464^
Sulforaphane	HDAC inhibitor	Inhibiting miR-19-mediated Wnt activity	Lung cancer (A549, H1299)^465^
Periplocin	Cardiotonic Steroid	Reducing TCF affinity to DNA	CRC (SW480)^466^
Henryin	Folk medicine to prevent GI disease	Blocking βcat binding to TCF4	CRC (HCT116)^467^
Cardamonin	TRP channel inhibitor	Inhibiting Akt which phosphorylates GSK3β	BC (MCF-7, BT-549, MDA-MB-231)^468^
Indirubin	Bacterial metabolism byproduct	Inhibiting GSK3β	Glioma^469^
Dihydroartemisinin	Anti-malaria drug	Unknown	SCC (A431)^470^
Shizukaol D	Algae extracts	Unknown	HCC (SMMC-7721, SK-HEP1, HepG2)^471^
Capsaicin	Pain relief drug	Inhibiting Tcf transcriptional activity	CRC (SW480, LoVo, HCT116)^472^
Carnosic acid		Blocking βcat binding to BCL9	CRC (SW480, HCT116)^473^
Ursolic acid		Enhancing phosphorylated GSK3β	PC (PC-3)^474^
New insights			
Niclosamide	Anthelmintic drug	Promoting Fzd1 endocytosis	OS (U2OS)^404^
		Interfering βcat-Tcf/Lef	CRC (HT29, HCT116, CaCO2)^475,476^
		Unknown	OC (SKOV3)^477^
		Inducing Lrp6 degradation	PC (PC-3, DU-145)^478^; BC (MDA-MB-231, T-47D)^478^
Pimozide	Psychotropic drug	Unknown	HCC (Hep3B, HepG2)^479^
		Unknown	CRC (SW480, HCT116)^480^
Ethacrynic acid	Diuretic drug	Inhibiting the recruitment of Lef1	CLL^481,482^
		Unknown	HCC (Hep3B, HepG2)^483^
Pyrvinium	Antiparasitic drug	Activating CK1α,^194^ or maybe GSK3β^361^	CRC (HCT116, SW480)
Salinomycin	Antimicrobial agent	Inducing Lrp6 degradation	BC (HS578T, MDA-MB-231)^406^; PC (PC-3, DU-145)^406^
		Unknown	HNSCC (CNE-1, CNE-2, CNE-2/DDP)^484^
		Increasing intracellular Ca^2+^ level	HCC (HepG2, BEL-7402)^485^
Sulindac	NSAID	Inhibiting βcat transcriptional activity	CRC (SW620, HT-29)^486,487^
		Inhibiting βcat transcriptional activity	NSCLC (A549); BC (MCF-7)^487^
Celecoxib	NSAID	Promoting βcat degradation by inhibiting E2 synthesis	BC (MCF-7 and MDA-MB-231)^488^
		Caspase-mediated βcat degradation^489^	CRC (HCT-116, HT29, DLD-1)^490^
TAK-715 and AMG-548	P38 inhibitor	Inhibiting CK1δ, ε	OS (U2OS)^491^
BBI608	STAT3 inhibitor	Unknown	CRC^373^
Lithium chloride		Competing with Mg^2+^ to inhibit Gsk3	Esophageal cancer (Eca-109)^492^
		Activating AKT to inhibit Gsk3	MLL^493^
Dovitinib	RTK inhibitor	Inhibiting the interaction of TNIK with ATP and TCF4	Multiple myeloma (IM-9)^494^
Streptonigrin	Antimicrobial agent	Blocking TCF binding to DNA	CRC (SW480)^495^

*ACC* adrenocortical carcinoma, *AML* acute monocytic leukemia, *BC* breast cancer, *CLL* chronic lymphocytic leukemia, *CRC* colorectal cancer, *HC* hepatoma carcinoma, *HNSCC* head and neck squamous cell carcinoma, *HL* Hodgkin lymphoma, *MCL* mantle cell lymphoma, *MM* multiple myeloma, *NSCLC* non-small cell lung cancer, *OS* osteosarcoma, *PC* prostate cancer, *SS* synovial sarcoma, *βcat* β-catenin

## Challenges of Wnt/β-catenin-targeted therapies

Even though drug development in the field of Wnt targeted therapies in cancers has been so prosperous, no exclusive drug has yet been approved by the FDA. This dilemma has aroused an un-neglectable crisis for moving forward the targeted therapies of canonical Wnt signaling in cancers. There are still many challenges before they could be put into large-scale clinical trials. This review crudely divided these drugs into three categories: SMIs, mAbs, and modified peptides. The molecular and structural properties determine the potential diversity of the clinical applications of these three categories. mAbs and peptidomimetics are more suitable for the targets on cell membrane surface, accordingly, SMIs are more suitable for targeting Wnt receptors and intracytoplasmic kinases, and stapled peptides are suitable for disrupting PPI in the nucleus.

For SMI, they are known for their good permeability and oral bioavailability, but off-target effects are common. This is especially inevitable for ATP-competitive drugs. mAbs are known for their unparalleled specificity, but their oral availability is poor. The mAbs have a slow clearance rate and a long half-life, which increases the burden on the liver and kidneys of the patient and increases the side effects. Besides, mAbs also have poor permeability and cannot target intracellular sites. Most importantly, current modifications of mAbs failed to decrease the antigenicity and increase tolerance. For modified peptides, they can achieve good permeability and tolerability, but a larger dose is required for them to maintain the therapeutic concentration.

Considering the ubiquitous and extensive involvements of Wnt/β-catenin signaling in regulating various normal tissue functions, it is ineluctable to accept the unintended byproducts of targeting canonical Wnt signaling. Although it provides an ideal approach to target nuclear β-catenin in aberrant canonical Wnt signaling-associated cancers, it is currently unprocurable to develop such kinds of targeted agents because of the very limited druggability. This crisis of developing the targeted therapies of Wnt/β-catenin signaling in cancers may possibly be overcome via the in-depth analysis of the preexisting molecules. Also, more development strategies such as immunotherapy, the synergistic medications of drugs, the decrement of mAbs weight, and so on could endow us with underlying breakthroughs to realize the canonical Wnt signaling targeted therapies in cancers.

## Conclusion and discussion

**Fig. 5:**
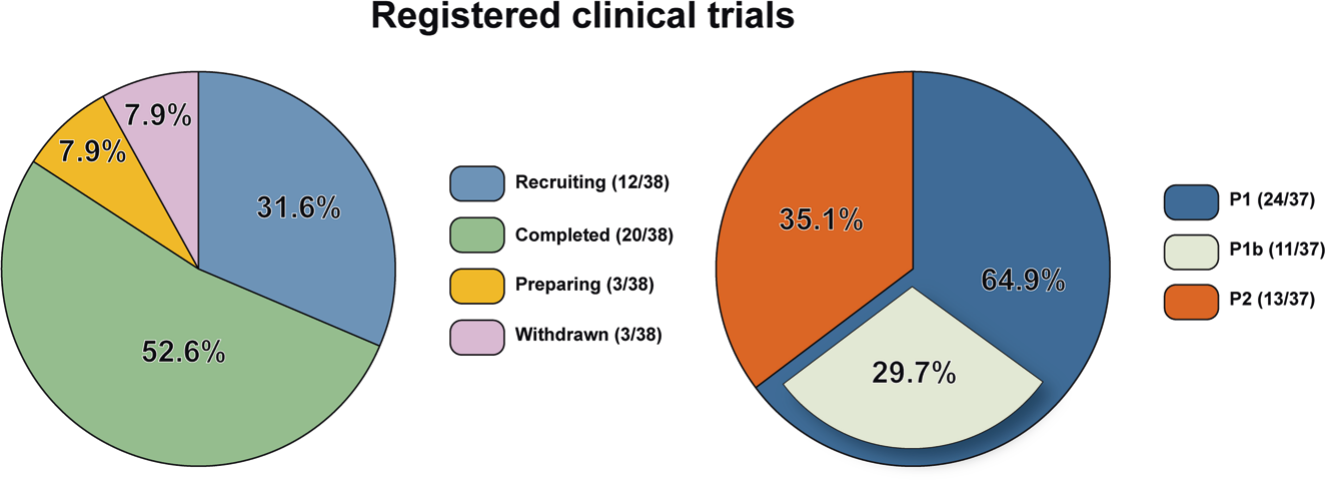
The statistical summary of up-to-date registered clinical trials of targeted therapies via targeting Wnt/β-catenin signaling in cancers. The left panel indicated the stages and the right one showed the phases of clinical trials. P1 phase I, P1b phase IB, P2 phase 2. All clinical trials included were updated until May 2021

Identified decades ago, Wnt/β-catenin signaling immediately generated substantial interest in the field of cancer research because of their extensive involvements and intensive roles in regulating numerous aspects of cancers, including the initiation, development, progression, diagnosis. In this study, we revealed that the status quo investigations have depicted an un-neglectable crisis of the Wnt/β-catenin-dependent targeted therapies in cancers. In detail, most of the medications targeting Wnt/β-catenin signaling in cancers have not been enrolled into clinical trials, and even the registered clinical trials remain in the very early phases of which most have not demonstrated satisfactory outcomes (Fig. 5). However, this dilemma clashed with the early evidence in in vitro and in vivo with the optimal performance of targeting Wnt/β-catenin signaling in cancers. The reasons for these poor therapeutic benefits rely on the fact that the current remedies of Wnt/β-catenin signaling-associated targeted therapies in cancers often lack satisfactory efficacy, specificity, and safety. For instance, due to the crucial roles of Wnt/β-catenin signaling in bone, many targeted therapies demonstrated obvious side effects of severe bone loss. Furthermore, certain SMIs and mAbs targeting Wnt/β-catenin signaling showed limited specificity because of the difficulty of identifying the druggable structures and sites of Wnt/β-catenin signaling components. These facts suggest that Wnt/β-catenin signaling targeted therapies in cancers are still lagging behind for a solid clinical translation.

**Fig. 6:**
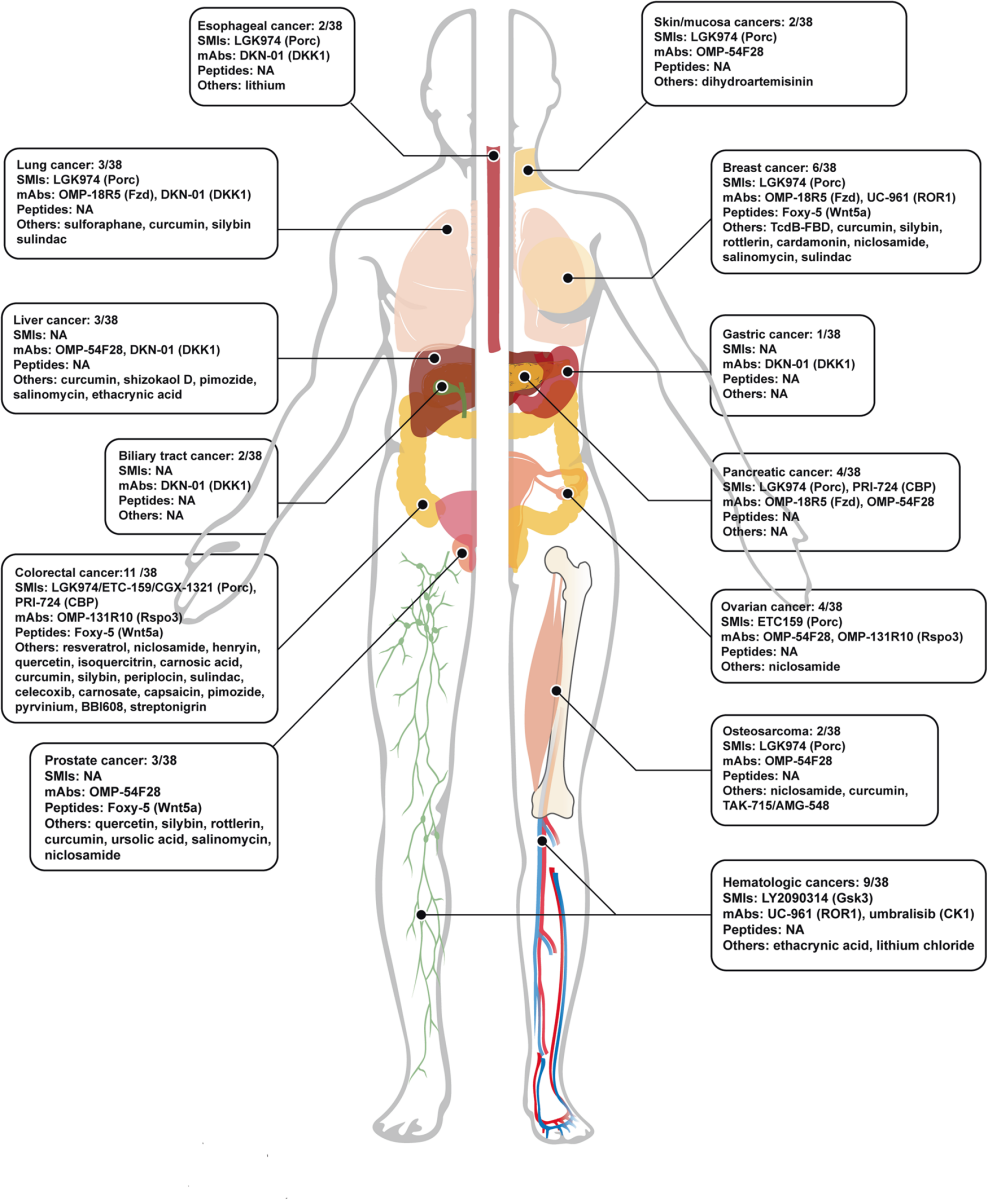
The overview of status quo registered clinical trials of Wnt/β-catenin signaling-dependent targeted therapies in cancers (updated in May 2021). Within the boxed diagram, the first lane provided the numbers of clinical trials for the specific type of cancers in all clinical trials. From the second to the fifth lane, we provided the types of drugs as SMIs, mAbs, Peptides, and others, and the parts that follow the colon indicted the names of drugs and the targeted components of Wnt/β-catenin signaling were shown in the brackets

Nevertheless, as massive early experimental investigations have already proven the benefits of targeting Wnt/β-catenin signaling in cancers it is still worthy to further analyze the capabilities of developing Wnt/β-catenin signaling targeted therapies in the future (Fig. 6). It should be noticed that future studies are called to solve the current crisis via decreasing the side effects but improving the specificity and safety of Wnt/β-catenin signaling targeted therapies in cancers. To sum up, our review systematically exhibited the strengths and weaknesses of the most updated targeted therapies of Wnt/β-catenin signaling in cancers, aiming to generate a thorough awareness of current challenges and crises. Ultimately, this study sought to provide future studies with the issues and insights that should be taken into account for developing better-targeted therapies of Wnt/β-catenin signaling in cancers.
